# Supplementary material for: An architectonic type principle in the development of laminar patterns of cortico-cortical connections
Source: Brain Struct Funct. 2021 Feb 9;226(4):979–87. doi: 10.1007/s00429-021-02219-6 (PMC8036174; doi:10.1007/s00429-021-02219-6)
Supplement: Supplementary file 1 — Supplementary file1 (DOCX 162 KB) [file 429_2021_2219_MOESM1_ESM.docx]

# Online Resources

Supplementary material for **An architectonic type principle in the development of laminar patterns of cortico-cortical connections** by Sarah F. Beul, Alexandros Goulas, Claus C. Hilgetag

Brain Structure and Function, <https://doi.org/10.1007/s00429-021-02219-6>

## Online Resource 1. Supragranular contribution to cortico-cortical connections in the immature macaque cortex.

| Immature *N*_SG_% vs. adult *N*_SG_% | Source area | Target area | Immature *N*_SG_% | Adult *N*_SG_% |
| --- | --- | --- | --- | --- |
| Neonatal *N*_SG_% [K89] |  |  |  |  |
|  | STS | V1 | 0.36 | 0.05 |
|  | V2 | V1 | 0.68 | 0.53 |
|  | V4 | V1 | 0.63 | 0.24 |
| Fetal *N*_SG_% [B02] |  |  |  |  |
|  | V1 | V4 | 0.85 | 1.00 |
|  | V2 | V4 | 0.96 | 0.95 |
|  | V3A | V4 | 0.73 | 0.63 |
|  | V5/MT | V4 | 0.65 | 0.56 |
|  | FST | V4 | 0.47 | 0.11 |
|  | LIP | V4 | 0.55 | 0.17 |
|  | TEO | V4 | 0.69 | 0.30 |
|  | TE | V4 | 0.43 | 0.29 |
|  | TH-TF | V4 | 0.06 | 0.002 |
|  | FEF | V4 | 0.73 | 0.75 |

| Enucleated *N*_SG_% vs. intact *N*_SG_% | Source area | Target area | Enucleated infant *N*_SG_% [M18] | Intact adult *N*_SG_% [C15] |
| --- | --- | --- | --- | --- |
|  | Entorhinal | V2 | 0.00 | -/- |
|  | FEF | V2 | 0.33 | -/- |
|  | FST | V2 | 0.14 | 0.07 |
|  | LIP | V2 | 0.37 | 0.05 |
|  | MST | V2 | 0.08 | 0.02 |
|  | V5/MT | V2 | 0.60 | 0.27 |
|  | Peri | V2 | 0.02 | 0.04 |
|  | PGa | V2 | 0.22 | 0.02 |
|  | PIP | V2 | 0.33 | 0.01 |
|  | STP | V2 | 0.15 | 0.16 |
|  | Subiculum | V2 | 0.00 | -/- |
|  | TE | V2 | 0.06 | 0.02 |
|  | TEO | V2 | 0.39 | 0.09 |
|  | TH/TF | V2 | 0.00 | 0.01 |
|  | V1 | V2 | 1.00 | 0.74 |
|  | V3 | V2 | 0.59 | 0.32 |
|  | V3A | V2 | 0.49 | 0.03 |
|  | V4 | V2 | 0.41 | 0.25 |
|  | 7A | V4 | 0.05 | 0.04 |
|  | FEF | V4 | 0.44 | -/- |
|  | FST | V4 | 0.20 | 0.17 |
|  | LIP | V4 | 0.24 | 0.22 |
|  | MST | V4 | 0.16 | 0.04 |
|  | V5/MT | V4 | 0.35 | 0.46 |
|  | Peri | V4 | 0.00 | 0.0004 |
|  | PIP | V4 | 0.44 | 0.15 |
|  | STP | V4 | 0.04 | 0.02 |
|  | TE | V4 | 0.05 | 0.09 |
|  | TEO | V4 | 0.45 | 0.43 |
|  | TH/TF | V4 | 0.05 | 0.01 |
|  | V1 | V4 | 0.00 | 0.98 |
|  | V2 | V4 | 0.83 | 0.93 |
|  | V3 | V4 | 0.65 | 0.66 |
|  | V3A | V4 | 0.63 | 0.00 |

Values for B02 extracted from their Figure 7A. K89: Kennedy et al., 1989; B02: Batardière et al., 2002; M18: Magrou et al., 2018; C15: Chaudhuri et al., 2015.

## Online Resource 2. Correlations of laminar projection patterns and architectonic differentiation in the immature macaque cortex.

|  | *ρ* | *p* value | R² |
| --- | --- | --- | --- |
| Adult *N*_SG_% vs. immature *N*_SG_% |  |  |  |
| Neonate (K89) | 1.00 | 0.3333 | 0.77 |
| Fetal (B02) | 0.94 | 0.0000 | 0.75 |
| Immature *N*_SG_% vs. architectonic type difference |  |  |  |
| Neonate (K89) | 1.00 | 0.3333 | 0.95 |
| Fetal (B02) | 0.87 | 0.0011 | 0.74 |
| Adult *N*_SG_% vs. architectonic type difference |  |  |  |
| Neonate (K89) | 1.00 | 0.3333 | 0.92 |
| Fetal (B02) | 0.87 | 0.0009 | 0.74 |
| Immature *N*_SG_% vs. neuron density difference |  |  |  |
| Neonate (K89) | 1.00 | 0.3333 | 0.82 |
| Fetal (B02) | 0.73 | 0.0311 | 0.37 |
| Adult *N*_SG_% vs. neuron density difference |  |  |  |
| Neonate (K89) | 1.00 | 0.3333 | 1.00 |
| Fetal (B02) | 0.72 | 0.0369 | 0.54 |
| Adult *N*_SG_%/immature *N*_SG_% vs. architectonic type difference |  |  |  |
| Neonate (K89) | 1.00 | 0.3333 | 0.91 |
| Fetal (B02) | 0.77 | 0.0093 | 0.62 |
| Adult *N*_SG_%/immature *N*_SG_% vs. neuron density difference |  |  |  |
| Neonate (K89) | 1.00 | 0.3333 | 0.99 |
| Fetal (B02) | 0.58 | 0.1080 | 0.38 |

See Figure 1 for scatter plots of the underlying data. *ρ* and *p* value: Spearman rank-correlation; R²: coefficient of determination for a linear regression model. Please note that the *p* value for K89 correlations cannot be lower than 0.33 because only three data points are available.

## Online Resource 3. Correlations of laminar projection patterns and architectonic differentiation in the immature macaque cortex for alternative assignments of cortical areas to region STS

In the main text, we matched the projection data of Kennedy et al. (1989) for the cortical region STS to areas FST, PGa, and STPi in the M132 parcellation of Markov et al. (2014). Prompted by the comments of one reviewer of this paper, here we consider alternative assignments of cortical areas of the Markov et al. (2014) M132 parcellation to region STS, first area V5/MT by itself, and second are V5/MT together with area FST. The respective resulting correlations and alternative figures to Figure 1 of the main text are shown below. These results show that the exact assignment (STS vs. V5/MT vs. V5/MT +FST) does not result in a fundamental difference to the principal findings of the main text. The reason for why the alternative results do not differ very much is that these areas are of a relatively similar structural type (V5/MT=type 6; FST=type 4; STP=type 4; PG(A7a)=type 4; cf. Hilgetag et al, 2016), leading to only minor shifts in the findings.

### Assigning area V5/MT to STS

|  | *ρ* | *p* value | R² |
| --- | --- | --- | --- |
| Adult *N*_SG_% vs. immature *N*_SG_% |  |  |  |
| Neonate (K89) | 1.00 | 0.3333 | 0.77 |
| Fetal (B02) | 0.94 | 0.0000 | 0.75 |
| Immature *N*_SG_% vs. architectonic type difference |  |  |  |
| Neonate (K89) | 0.87 | 0.6667 | 0.39 |
| Fetal (B02) | 0.87 | 0.0011 | 0.74 |
| Adult *N*_SG_% vs. architectonic type difference |  |  |  |
| Neonate (K89) | 0.87 | 0.6667 | 0.85 |
| Fetal (B02) | 0.87 | 0.0009 | 0.74 |
| Immature *N*_SG_% vs. neuron density difference |  |  |  |
| Neonate (K89) | 1.00 | 0.3333 | 0.54 |
| Fetal (B02) | 0.73 | 0.0311 | 0.37 |
| Adult *N*_SG_% vs. neuron density difference |  |  |  |
| Neonate (K89) | 1.00 | 0.3333 | 0.94 |
| Fetal (B02) | 0.72 | 0.0369 | 0.54 |
| Adult *N*_SG_%/immature *N*_SG_% vs. architectonic type difference |  |  |  |
| Neonate (K89) | 0.87 | 0.6667 | 0.87 |
| Fetal (B02) | 0.77 | 0.0093 | 0.62 |
| Adult *N*_SG_%/immature *N*_SG_% vs. neuron density difference |  |  |  |
| Neonate (K89) | 1.00 | 0.3333 | 0.95 |
| Fetal (B02) | 0.58 | 0.1080 | 0.38 |

See Alternative Figure 1 below for scatter plots of the underlying data. *ρ* and *p* value: Spearman rank-correlation; R²: coefficient of determination for a linear regression model. Please note that the *p* value for K89 correlations cannot be lower than 0.33 because only three data points are available.

##
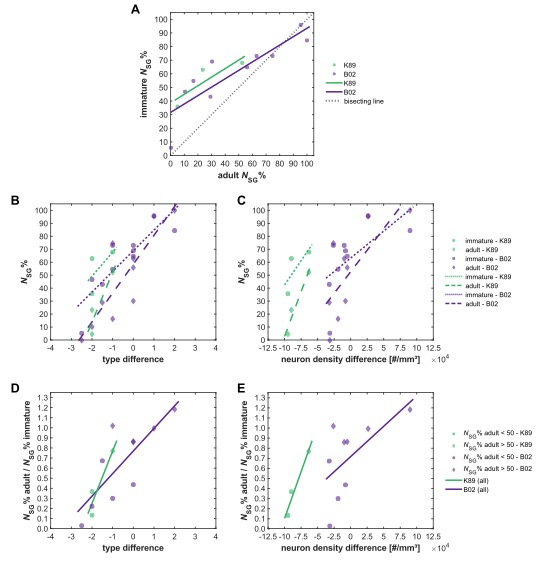


## Alternative Figure 1. Laminar projection patterns in immature cortex.

Relative contribution of supragranular projection neurons (*N*_SG_%) to projections targeting areas V1 (K89, neonatal) and V4 (B02, fetal) in the immature macaque cortex. (A) Immature *N*_SG_% in relation to the corresponding adult *N*_SG_%. (B) *N*_SG_% for both immature and adult cortex in relation to architectonic differentiation measured as difference in architectonic type, where type difference = type_source area_− type_target area_. (C) *N*_SG_% for both immature and adult cortex in relation to architectonic differentiation measured as difference in neuron density, where neuron density difference = density_source area_− density_target area_. (D) Fraction of supragranular projection neurons observed in the immature cortex that remains in the adult cortex in relation to difference in architectonic type. (E) Fraction of supragranular projection neurons observed in the immature cortex that remains in the adult cortex in relation to difference in neuron density. In these data, the supragranular contribution declines with maturation. That is, in (D) and (E), the value of adult *N*_SG_% divided by immature *N*_SG_% is below 1 for most areas. Projection data from K89 (Kennedy et al. 1989) and B02 (Batardière et al. 2002).

### Assigning areas V5/MT and FST to STS

|  | *ρ* | *p* value | R² |
| --- | --- | --- | --- |
| Adult *N*_SG_% vs. immature *N*_SG_% |  |  |  |
| Neonate (K89) | 1.00 | 0.3333 | 0.77 |
| Fetal (B02) | 0.94 | 0.0000 | 0.75 |
| Immature *N*_SG_% vs. architectonic type difference |  |  |  |
| Neonate (K89) | 1.00 | 0.3333 | 0.86 |
| Fetal (B02) | 0.87 | 0.0011 | 0.74 |
| Adult *N*_SG_% vs. architectonic type difference |  |  |  |
| Neonate (K89) | 1.00 | 0.3333 | 0.98 |
| Fetal (B02) | 0.87 | 0.0009 | 0.74 |
| Immature *N*_SG_% vs. neuron density difference |  |  |  |
| Neonate (K89) | 1.00 | 0.3333 | 0.54 |
| Fetal (B02) | 0.73 | 0.0311 | 0.37 |
| Adult *N*_SG_% vs. neuron density difference |  |  |  |
| Neonate (K89) | 1.00 | 0.3333 | 0.94 |
| Fetal (B02) | 0.72 | 0.0369 | 0.54 |
| Adult *N*_SG_%/immature *N*_SG_% vs. architectonic type difference |  |  |  |
| Neonate (K89) | 1.00 | 0.3333 | 0.98 |
| Fetal (B02) | 0.77 | 0.0093 | 0.62 |
| Adult *N*_SG_%/immature *N*_SG_% vs. neuron density difference |  |  |  |
| Neonate (K89) | 1.00 | 0.3333 | 0.95 |
| Fetal (B02) | 0.58 | 0.1080 | 0.38 |

See Alternative Figure 1 below for scatter plots of the underlying data. *ρ* and *p* value: Spearman rank-correlation; R²: coefficient of determination for a linear regression model. Please note that the *p* value for K89 correlations cannot be lower than 0.33 because only three data points are available.


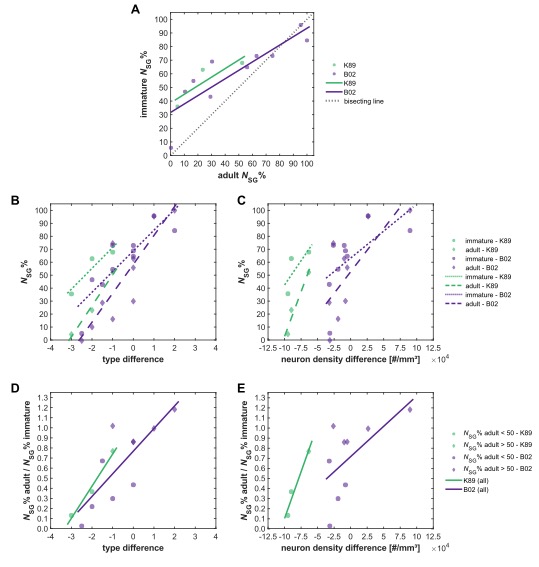


## Alternative Figure 1. Laminar projection patterns in immature cortex.

Relative contribution of supragranular projection neurons (*N*_SG_%) to projections targeting areas V1 (K89, neonatal) and V4 (B02, fetal) in the immature macaque cortex. (A) Immature *N*_SG_% in relation to the corresponding adult *N*_SG_%. (B) *N*_SG_% for both immature and adult cortex in relation to architectonic differentiation measured as difference in architectonic type, where type difference = type_source area_− type_target area_. (C) *N*_SG_% for both immature and adult cortex in relation to architectonic differentiation measured as difference in neuron density, where neuron density difference = density_source area_− density_target area_. (D) Fraction of supragranular projection neurons observed in the immature cortex that remains in the adult cortex in relation to difference in architectonic type. (E) Fraction of supragranular projection neurons observed in the immature cortex that remains in the adult cortex in relation to difference in neuron density. In these data, the supragranular contribution declines with maturation. That is, in (D) and (E), the value of adult *N*_SG_% divided by immature *N*_SG_% is below 1 for most areas. Projection data from K89 (Kennedy et al. 1989) and B02 (Batardière et al. 2002).

## Online Resource 4. Correlations of laminar projection patterns after enucleation.

|  | All data points | | | V1 excluded | | |
| --- | --- | --- | --- | --- | --- | --- |
|  | *ρ* | *p* value | R² | *ρ* | *p* value | R² |
| *N*_SG_% enucleated vs. *N*_SG_% intact | 0.47 | 0.0083 | 0.27 | 0.58 | 0.0013 | 0.48 |
| *N*_SG_% enucleated vs. structural type difference | 0.73 | 1.2e-6 | 0.44 | 0.86 | 4.2e-10 | 0.62 |
| *N*_SG_% enucleated vs. neuron density difference | 0.48 | 0.0185 | 0.21 | 0.60 | 0.0033 | 0.47 |

See Figure 2 for scatter plots of the underlying data. *ρ* and *p* value: Spearman rank-correlation; R²: coefficient of determination for a linear regression model. Projections originating in V1 were excluded because V1 was affected very strongly by the enucleation and the resulting *N*_SG_%-values are outliers.
